# Recent Advances in Device Engineering and Computational Analysis for Characterization of Cell-Released Cancer Biomarkers

**DOI:** 10.3390/cancers14020288

**Published:** 2022-01-07

**Authors:** Hesam Abouali, Seied Ali Hosseini, Emma Purcell, Sunitha Nagrath, Mahla Poudineh

**Affiliations:** 1 Department of Electrical and Computer Engineering, University of Waterloo, Waterloo, ON N2L 3G1, Canada; hesam.abouali@uwaterloo.ca (H.A.); sahosseini.ee@gmail.com (S.A.H.); 2 Department of Chemical Engineering, University of Michigan, Ann Arbor, MI 48109-2800, USA; eapurcel@umich.edu (E.P.); snagrath@umich.edu (S.N.)

**Keywords:** circulating tumor DAN, extracellular vesicles, clinical applications, technologies, computational methods

## Abstract

**Simple Summary:**

The circulating cancer biomarkers, known as ‘liquid biopsy’ (LB), represent a means to profile tumors non-invasively and collect information that can define therapeutic regimens for precision and personalized medicine. Various approaches have been developed for isolating and studying the individual circulating cancer biomarkers. This review focuses on LB biomarkers of circulating tumor DNA (ctDNA) and small Extracellular vesicles (sEVs). We present the most recent approaches for their isolation and characterization and elaborate on the emerging mathematical and computational models for studying the roles of these cell-released cancer biomarkers in cancer progression. We envision that the study of these new models and technologies could significantly contribute to the field of personalized medicine.

**Abstract:**

During cancer progression, tumors shed different biomarkers into the bloodstream, including circulating tumor cells (CTCs), extracellular vesicles (EVs), circulating cell-free DNA (cfDNA), and circulating tumor DNA (ctDNA). The analysis of these biomarkers in the blood, known as ‘liquid biopsy’ (LB), is a promising approach for early cancer detection and treatment monitoring, and more recently, as a means for cancer therapy. Previous reviews have discussed the role of CTCs and ctDNA in cancer progression; however, ctDNA and EVs are rapidly evolving with technological advancements and computational analysis and are the subject of enormous recent studies in cancer biomarkers. In this review, first, we introduce these cell-released cancer biomarkers and briefly discuss their clinical significance in cancer diagnosis and treatment monitoring. Second, we present conventional and novel approaches for the isolation, profiling, and characterization of these markers. We then investigate the mathematical and in silico models that are developed to investigate the function of ctDNA and EVs in cancer progression. We convey our views on what is needed to pave the way to translate the emerging technologies and models into the clinic and make the case that optimized next-generation techniques and models are needed to precisely evaluate the clinical relevance of these LB markers.

## 1. Clinical Significance of Cell-Released Cancer Biomarkers

**Figure 1:**
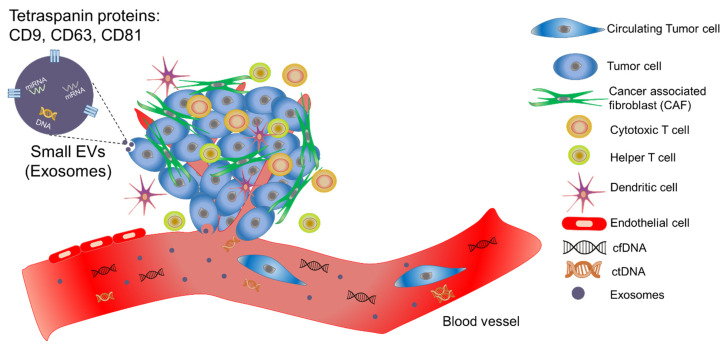
A tumor microenvironment consists of a heterogeneous mixture of cellular and non-cellular components alongside cancerous cells.

The tumor microenvironment (TME) comprises a heterogeneous mixture of cellular and non-cellular components, including cancer cells, fibroblasts, immune cells, blood vessels, and intercellular signaling factors (Figure 1). It contributes to the maintenance of cancer stemness, and mounting evidence indicates that it plays a critical role in treatment resistance, uncontrolled tumor growth, promoting angiogenesis, invasion, and metastasis.

**Figure 2:**
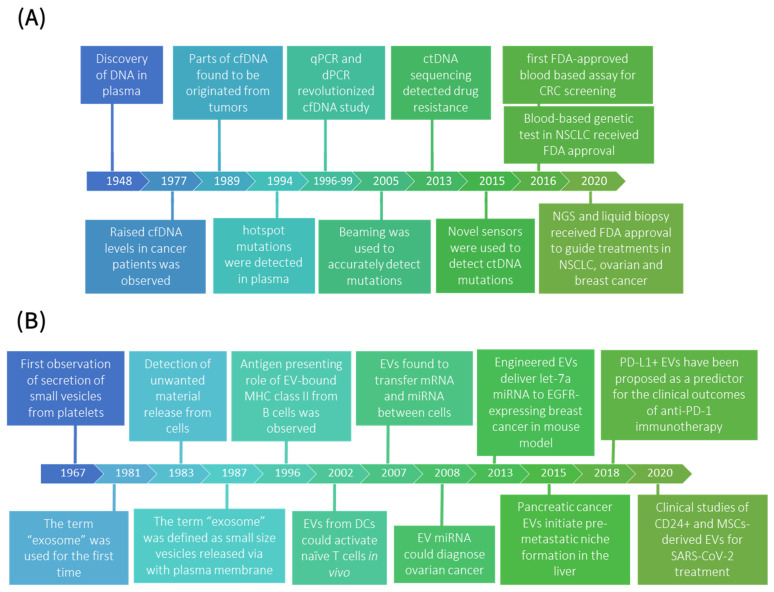
Timeline of significant discoveries related to (**A**) ctDNA [2,3,19,20,21,22,23,24,25,26,27,28] and (**B**) small EVs’ (exosomes’) [40,41,42,43,44,45,46,47,48,49,50,51,52,53,54,55] biology, to their clinical significance, and to technologies that have advanced their clinical utility.

Liquid biopsy (LB) biomarkers, including circulating tumor cells (CTCs), tumor-educated platelets, extracellular vesicles (EVs), circulating cell-free DNA (cfDNA), and circulating tumor DNA (ctDNA), are released into the blood stream during cancer progression in TME. In recent years, LB has emerged as a viable alternative to tissue genotyping for the assessment of tumor-specific molecular alterations in cancer patients. Among LB biomarkers, ctDNA and tumor-derived EVs are the ones that are released from cancerous cells. ctDNA is mainly released by apoptotic or necrotic tumor cells [1,2] while cfDNA originates both from tumor and non-tumor cells of the TME, as well as cells from other parts of the body [3]. Elevated cfDNA fragment concentrations have been observed in the blood of cancer patients and in those with metastatic disease compared to non-metastatic patients, suggesting that this metric may be useful for diagnosis [4]. cfDNA levels were used as a diagnostic tool for colorectal [5,6], endometrial [7,8], ovarian [9], breast [10], melanoma [11], oropharyngeal [12], pancreatic [13], prostate [14,15], and non-small-cell lung cancers (NSCLC) [16,17,18]. However, to precisely predict tumor behavior, assess treatment efficacy, and determine mutational load, it is necessary to determine the proportion of aberrant vs. wild-type DNA and thoroughly characterize the tumor-derived DNA [19]. This was not possible prior to the invention of digital polymerase chain reaction (dPCR) [20] and real-time PCR [21], which assisted researchers in accurately quantifying mutant genes for applications in diagnosis [19] and prognosis [22]. Figure 2A underlines the most important discovery related to cfDNA and ctDNA [3,23,24,25,26,27,28].

Several studies have confirmed that ctDNA carries mutation and methylation profiles similar to those of their tumors of origin [29,30]. The FDA has approved a number of mutations for cancer screening or cancer assaying, including mutations in the *BRCA1* and *BRCA2* genes for ovarian cancer, *ALK* rearrangements for NSCLC, and alterations in the *PIK3CA* gene for breast cancer patients [28]. Tissue-specific methylation patterns recovered from ctDNA could also reveal the tumor location and help in cancer screening [31,32,33]. For example, SEPT9 is a methylation pattern that has recently received FDA approval as the first tumor biomarker to screen metastatic colorectal cancer (mCRC) and may be assessed as a staging biomarker [31,34]. The detection of mutations and epigenetic changes in ctDNA can thus guide physicians toward efficient therapies that can defeat the drug resistance of tumors [35]. Moreover, the detection of ctDNA mutations could alert clinicians to the presence of minimal disease residues or micro-metastases in patients with seemingly successful initial treatment, which could result in subsequent cancer relapse at distant sites [36]. Although the applications of cfDNA and ctDNA as LB biomarkers have accelerated over the past decade, especially for screening patients with NSCLC and CRC, larger clinical studies are still needed to fully discover their roles. Advancements in technologies to capture and characterize plasma DNA with high sensitivity and selectivity will aid this endeavor.

EVs are micro/nanoscale structures with a size ranging from 30 to 1000 nm that are released from the cell, delimited by a lipid bilayer, and cannot replicate, i.e., do not contain a functional nucleus [37,38]. Exosomes with sizes of less than 200 nm, which were recently re-named small EVs or sEVs by new international society for extracellular vesicles guideline [38], have been used in various cancer diagnostic applications, from molecular monitoring and disease staging to dynamic cancer therapy and progression monitoring. A critical benefit of sEVs compared to ctDNA is their various cargoes; where ctDNA is restricted to DNA analyses, sEVs carry an array of cargoes, including RNA, DNA, proteins, and lipids [39]. Identifying specific molecular targets within sEVs will allow clinicians to monitor patients non-invasively while also providing highly personalized treatment. Figure 2B highlights the significant discoveries related to the sEVs, from their biology to their clinical importance [40,41,42,43,44,45,46,47,48,49,50,51,52,53,54]. RNA is the most commonly profiled sEV marker for prognostic and treatment monitoring applications with specific miRNA signatures being predictive of worse survival in lung [55], liver [56,57], colorectal [58], glioma [59], and prostate [60] cancers. miRNA abundance and expression have also been found to be diagnostic compared to healthy controls; lung cancer patients had dramatically higher miRNA expression [61] and unique signatures [62] compared to healthy populations. Prognostic signatures of mRNA or other long RNA have been shown to be predictive of worse survival in several cancers, including NSCLC [63] and hepatocellular carcinoma [64]. Similarly, sEV proteins have been suggested for diagnostic and prognostic assays in many cancers, including lung [65,66], breast [67], and pancreatic [68] cancers.

In the TME, sEVs are a means of communication between cancerous cells and other cells and are uniquely biocompatible. Therefore, they can hypothetically target tumor cells and transfer drugs or other targets to kill them or prohibit their proliferation [69]. Many groups have developed novel sEV engineering solutions to deliver standard anti-tumor drugs, such as chemotherapies or immunotherapies [70]. For example, sEVs extracted from NSCLC cells were embedded with doxorubicin (DOX), a well-known chemotherapy drug, and conjugated with gold nanoparticles. These EV-based conjugates were used to deliver drugs to lung cancer cell lines and non-tumorigenic lung fibroblasts. Compared with free DOX delivery, the nanoscale EV–drug complex had higher toxicity against cancer cells, while the effects of DOX on healthy cells were reduced [71]. EVs have also been recently used to deliver novel therapeutics, including siRNA and CRISPR/Cas-9, to specifically induce cell death in vivo and in vitro, respectively [72,73].

To improve cancer detection and treatment, the molecular profiles gathered from ctDNA can be complemented with those obtained from tumor-derived sEVs that contain DNA and RNA. Thus, it is important to isolate and characterize these cell-released LB biomarkers with high sensitivity. In this review, we discuss recent advances in device engineering and computational analysis that enable ctDNA and sEV characterization.

## 2. Recent Technology Advances for Studying Cell-Released Biomarkers

The massive use of LBs as biomarkers for personalized cancer diagnosis and screening has led to the rapid development of novel technologies for their isolation and characterization. Technologies related to cfDNA/ctDNA and sEV capture have been reviewed [74,75,76,77,78,79]; for this reason, below, we only highlight the most advanced approaches (in the past two years) while introducing the conventional and gold standard techniques. Specifically, we categorize these advances into two groups: extraction and isolation technologies versus advancements in characterization.

### 2.1. Strategies for cfDNA/ctDNA Isolation and Characterization

#### 2.1.1. Conventional Methods for cfDNA/ctDNA Extraction

cfDNA is typically extracted using commercially available kits, which are either based on organic compounds that facilitate ion exchange binding [75] or solid-phase extraction (SPE) that utilizes silica beads/membrane as the binding site to DNA molecules in the presence of specific conditions such as high chaotropic salts concentrations and low pH solutions [80]. The cfDNA level is an important indicator of cancer progression and can be measured using real-time PCR [81]. While nucleic acid extraction from plasma using the conventional methods appears to be a straightforward procedure, there are challenges regarding the extraction of cfDNA without loss of its important subset: ctDNA. In fact, ctDNA molecules are shorter than non-tumor-derived cfDNA (150 to 200 base pairs (bps) compared to up to 10,000 bps for cfDNA), which along with their lower quantities, makes their isolation more difficult [82,83].

#### 2.1.2. Novel Strategies for cfDNA/ctDNA Extraction

**Figure 3:**
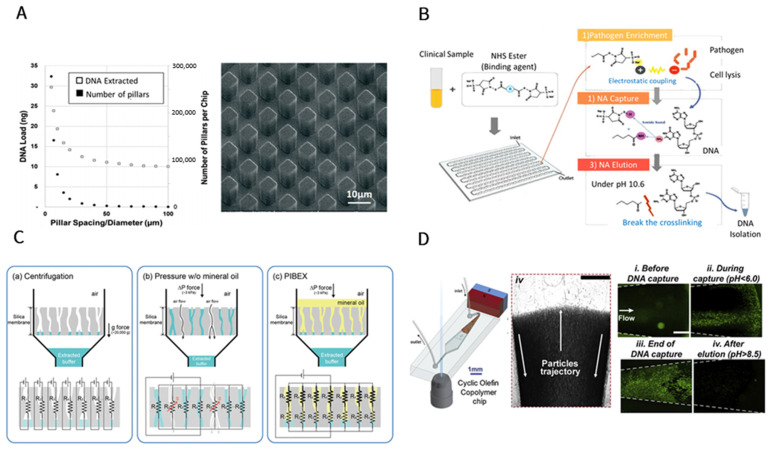
Novel Technologies to extract cfDNA/ctDNA. (**A**) The high surface area of micropillars in this plastic chip improves extraction yield of cfDNA. The Inner side surface of this low-cost chip is activated by UV/O_3_ and has been able to capture >90% of medium-size cfDNA (100–700 bps) and >50% of short cfDNA (50 bps) micropillars. Reproduced with permission from [82]. (**B**) In a novel microfluidic chip that contains helix-shaped microchambers to avoid forming bubbles, bis-(sulfosuccinimidyl)suberate reagent is used in lieu of chaotropic and pH modifier reagents to capture cfDNA while this device is capable of enriching pathogens in blood at the same time. Reproduced with permission from [86]. Copyright 2020 American Chemical Society. (**C**) Conventional solid-phase extraction requires vacuum or centrifugation instruments, whereas in this work, an immiscible fluid was applied by an automated microfluidic chip (PIBEX) to push plasma through the columns of extraction [88]. Reproduced from an open source. (**D**) Magnetic Beads that can bind to DNA strands are immobilized by an external magnet while serum that contains cfDNA is flowing: as a result of the good dispersion of beads in the chamber and the effective collision of DNA molecules with the beads, extraction is enhanced. Reproduced with permission from [89]. Copyright 2019 Elsevier.

In order to resolve concerns regarding loss of ctDNA, microfluidic-based techniques have been implemented for cfDNA isolation, in which the incorporation of microstructures, such as microbeads [84], micropillars [82], and microwells [85,86], enhances the cfDNA/ctDNA extraction performance and consequently shortens the processing time (Figure 3A,B). The integration of operations in the microfluidic chips has resulted in higher extraction performance. This was especially of great importance in the case of short strands of cfDNA as the conventional methods were able to separate only 40% of DNA molecules [87] while the microchips were able to recover up to 70% [82]. These novel technologies were investigated both for their higher capture rate and their feasibility and accessibility in point-of-care applications.

One major drawback of conventional SPE methods is their requirement for bulky associated instruments, such as vacuum pumps. To tackle this challenge, a microfluidic device that automates and facilitates delivery of the sample, which uses immiscible liquids such as mineral oil to improve liquid movements through silica membrane pores and enhances the DNA isolation process, is reported. The results showed that the recovery rate of DNA using this technique increased by about 5% (Figure 3C) [88].

Magnetic force in microfluidic chips could be adjusted to manipulate microparticles and maximize their provided surface area. In this endeavor, a microfluidic chamber was recently designed that produces a dispersed arrangement of microparticles with the aid of magnetic force and enhances the capture rate. This method was used as an extraction step in line with droplet dPCR (ddPCR) for the downstream analysis and showed high preservation of rare genetic data in the subsequent studies. This microfluidic device was able to capture up to 80% of cfDNA, which is better than the conventional isolation kits (Figure 3D) [89].

Other physical principles are under investigation to extract cfDNA from blood, and dielectrophoresis is the most pioneering one. In a study, a simple microfluidic chip consisting of only two channels was built to separate cfDNA by electrophoresis for optical quantification [90]. Dielectrophoretic forces were also used to extract cfDNA for downstream analysis [91]. Advancements in dielectrophoretic-based devices led to the manipulation and capture of DNA in nanoscale compartments in a microfluidic chip [92].

#### 2.1.3. Conventional Methods for ctDNA Characterization

Unfortunately, ctDNA has a similar structure to other cfDNA subtypes, making its isolation and detection difficult. Thus, instead of separating ctDNA from other types of nucleic acids, the whole genetic data in blood, i.e., cfDNA, is interrogated to find and quantify sequences that could be attributed to a tumor origin such as mutations. The ratio of the level of strands with a tumor signature to the other strands, the absolute quantity of the tumor-derived strands, and the type of mutations that these strands convey are considered as the criteria for clinicians in deciding the best approaches to treat patients. For this purpose, known genetic data of a specific cancer are tracked in plasma. The rare genetic events cannot be detected by conventional real-time PCR techniques and require more precise devices with single-molecule resolutions. In the last two decades, two methods (modified PCR methods—particularly digital PCR—and whole-genome sequencing) have become successful in detection of rare events in blood and boosted the significance of LB considerably [93,94,95]. The amplification refractory mutation system [96,97], bidirectional pyrophosphorolysis-activated polymerization [98], BEAMing [99], dPCR [36], and DNA sequencing [100] (all amplification-based methods) are routinely used for this purpose; however, they are tedious, expensive, and require specialized instrumentation. Another limitation to conventional targeted ctDNA profiling techniques is their cost (estimated at $1750 per patient, including the cost of sequencing a single tumor region, synthesis of a patient-specific panel, and profiling of plasma samples) [101]. Novel strategies can purposefully identify a point mutation instead of sequencing the whole genome, saving money and time.

#### 2.1.4. Novel Strategies for ctDNA Detection

**Figure 4:**
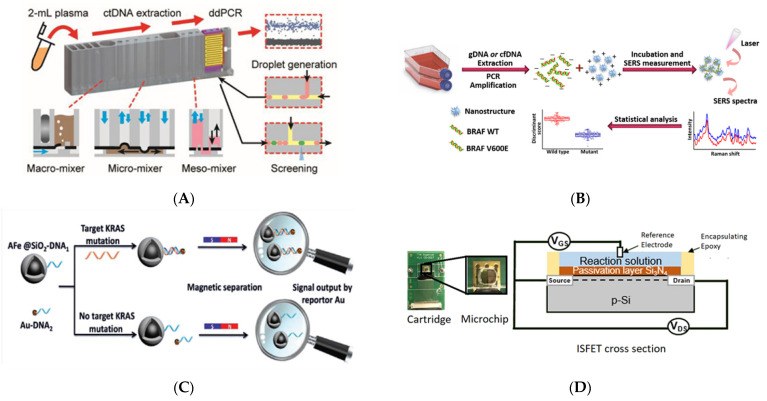
Novel Technologies to characterize ctDNA. (**A**) A miniaturized device extracts ctDNA from plasma first and then characterizes the extracted ctDNA by means of an integrated digital droplet PCR module. Reproduced with permission from [103]. Copyright 2020 American Chemical Society. (**B**) Shifts in surface-enhanced Raman spectroscopy (SERS) signals from DNA molecules that are entangled with plasmonic nanostructures were shown to be a sensitive and specific tool to reflect mutations in DNA molecules. Reproduced with permission from [107]. Copyright 2020 American Chemical Society. (**C**) Coupled Fe and Au nano-particles could effectively isolate ctDNA and detect the target mutation by means of single-stranded RNA probes with high sensitivity in a single step. Reproduced with permission from [108]. (**D**) The ISFET device could be used for the selective and rapid detection of mutations by monitoring the loop-based isothermal amplification of ctDNA samples [117]. Reproduced from an open source.

As ctDNA has a short half-life, the downstream analysis should be performed immediately after extraction [33], but there are many steps in the conventional methods requiring the handling of samples that raise the risk of contamination [102]. Hence, automation of this procedure inside the closed channels of microfluidic chips could minimize the risk of rare sample loss while reducing labor. Microfluidic devices are introduced for both extraction and analysis and demonstrate the benefit of integration in reducing operation time and enhancing the reliability of results. Such microdevices can detect rare mutations, such as the *T790M* mutation in *EGFR*, with a mutation-to-wild-type ratio of ~1% (Figure 4A) [103]. However, they cannot yet outperform the ddPCR systems, which can detect frequencies as low as 0.1% [104], and improvements are still needed in terms of sensitivity to detect rare mutations. It should be noted that such devices follow a procedure for the detection of mutations that is similar to that of conventional devices but occurs inside a miniaturized device. Miniaturization has the general benefits of lower reagent usage, increased mobility, and higher processing speed; however, for the improvement of sensitivity, other radical methods should be sought.

An approach that has been significantly successful in the reducing of processing time is the analysis of specific target mutations instead of whole sequencing of the sample, which may take up to several days. Particularly, Raman spectroscopy [105], surface plasmon resonance (SPR) [106], and electrochemical sensors have been proposed as the next-generation techniques and focus on the presence of the genetic data of interest taking only a few minutes to conclude. Direct assessment of DNA molecules for mutations instead of labeling the target is another distinctive advantage that is introduced alongside the use of Raman spectroscopy and is successfully applied to detect *BRAF* mutations in both genome and plasma DNA samples. In this work, the incorporation of plasmonic nanostructures and PCR amplification boosted the signal. The direct assessment approach showed the promise of high selectivity as the technique was capable of discrimination of a single-base variation in DNA molecules. However, the sensitivity of the device for detection of mutation (limit of detection = 100 copies of the DNA) was comparable to PCR-based methods and did not show a significant increase (Figure 4B) [107]. Therefore, these novel technologies have not been reported to show an increased sensitivity compared to the conventional devices despite the fact that they have reduced processing time and manual labor.

More interestingly, nanomaterials have been proposed for single-step mutation detection from plasma with a high sensitivity that obviates the need to extract DNA from samples and amplify the target allele. In this method, iron and gold nanoparticles connected to single-stranded nucleic acid probes were employed to isolate specific target mutations. Iron nanoparticles accomplished this through their magnetic properties, while gold nanoparticles detected ctDNA levels through inductively coupled plasma mass spectrometry. This method was also able to detect mutation-to-wild-type ratios that were as low as 0.12% and was found to be comparable to the ddPCR systems; however, the fact that this method does not require any bulky instruments makes it more facile (Figure 4C) [108].

Electrochemical approaches were among the very first methods applied for the detection of mutational hot spots [25,109,110,111] and methylation marks [112,113,114], attracting attention because of their high sensitivity, multiplexing ability, fast response, low cost, and simple setup [115]. These devices could be combined with isothermal methods for DNA amplification while keeping the system simple [116]. An ISFET (ion-sensitive field effect transistor) sensor that is mainly used for pH measurement as a chemical sensor was utilized to detect *PIK3CA* mutations by monitoring hydrogen ion generation during the loop-mediated isothermal amplification (LAMP) of DNA samples of breast cancer cell lines (Figure 4D) [117]. In electrochemical methods, electrodes are generally functionalized to capture and electrochemically report on ctDNA of interest. A challenge in this endeavor stems from the large panel of alterations that could occur in some genes such as EGFR, which can contain more than 40 mutations. To overcome this challenge, combinatorial probes were designed and successfully incorporated in electrodeposited gold nanoelectrodes to detect multiple mutations in EGFR using a single device [118,119]. The structures and materials of electrodes could have a strong effect and are examined in different shapes. Nanomaterials such as nanostructured gold microelectrodes [25,110,120], graphene-loaded iron oxide nanoparticles [119], and gold nanorods [106] have higher surface areas and have been used as modified electrodes to improve detection sensitivity. The results indicated that the limit of detection of this type of assay was as low as 0.01% for mutation-to-wild-type ratios, outperforming the competing ddPCR system.

**Outlook**. Overall, novel methods with high performance and affordable costs can pave the way for the clinical utility of ctDNA. Integration, direct assessment of point alterations in sequences, elimination of background wild-type alleles, and electrochemical measurement could be combined across the field of LB. It will be necessary to continue to simplify procedures by integrating the steps for sample preparation, ctDNA isolation, and characterization to eliminate extraneous equipment/steps. Up and coming technologies for cfDNA isolation are largely microfluidic devices, which provide higher efficiency and require less external equipment, allowing for the integration of the above steps into one device. However, these devices are incapable of processing large volumes of blood (>1 mL) and are sensitive to operational variables such as the external magnetic force or the perfusion flow rate. Furthermore, a preliminary plasma recovery step is usually required; therefore, cfDNA extractor devices that enable the processing of whole blood samples will deliver a significant advantage over conventional commercial kits. Following isolation, creative characterization methods save precious time by focusing on the detection of specific genetic data that are relevant to individual cancer types. Limitations include missing rare mutations in the detection panel and a dependency on instruments that are not conventional in clinical institutes such as plasma mass spectrometers or RAMAN detectors. We envision that addressing these challenges and performing rigorous validation and large cohort clinical trials will allow ctDNA to transform cancer care and study.

### 2.2. Strategies for sEV Isolation and Characterization

#### 2.2.1. Conventional Methods for sEV Isolation

The most widely used method is differential ultracentrifugation (UC) [121]; UC is commonly paired with a density gradient to yield higher purity [122]. Because of sample loss, sEV disruption, and the time-consuming nature of UC [123], this method has been optimized and integrated with filtration methods [124,125]. Size exclusion chromatography, which is also based on size differences, is used to isolate sEVs. Drawbacks to the technique, such as long operation time [78] and column variability [126], led researchers to optimize [126] or integrate it with UC [127] and ultrafiltration [128] methods. Other common isolation methods include commercial polymer precipitation methods that are used to precipitate and concentrate the sEVs [129]. Polymer precipitation methods benefit from relatively short processing times but are limited, however, by their throughput.

Immunoaffinity capture EV isolation was also implemented in commercial kits where EVs are captured on functionalized surfaces with capturing agents [130]. The most common isolation targets are the tetraspanins CD9, CD63, and CD81 or markers such as EpCAM. However, these targets result in bulk EV isolation, as they are commonly expressed across EV subtypes. With a similar approach, immunomagnetic methods use beads conjugated with capturing-antibodies to isolate EVs based on common surface biomarkers [131,132,133].

These methods are known to have different compatibility with downstream assays. For example, UC without a gradient was shown to have lower sEV yield but high purity when performing protein analyses compared with polymer precipitation methods [134]. Conversely, polymer precipitation kits have been shown to lead to high contamination but also high yield of small RNA compared to UC [122,134].

#### 2.2.2. New Strategies for EV Isolation

**Figure 5:**
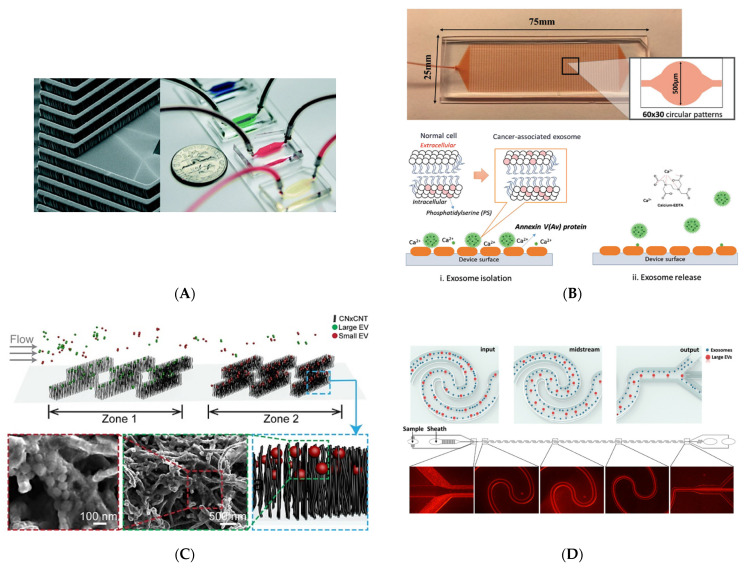
New strategies to isolate EVs. (**A**) SEM image of the fabricated herringbone structure and final devices. Inside of the devices, herringbone grooves enhance the possibility of an encounter between anti-CD9- and EpCAM-treated surfaces with EVs to isolate them. Reproduced with permission from [136]. (**B**) The design of the microfluidic device with circular regions, where Annexin V is coated to capture phosphatidylserine-positive EVs. To release the captured EVs, EDTA is used as the Ca^2+^ chelation agent. Reproduced with permission from [143]. (**C**) The size exclusion of EVs within the device with arrays of carbon nanotubes. Zones with different intratubular distances make the isolation of large and small EVs possible. Reproduced with permission from [147]. Copyright 2020 American Chemical Society. (**D**) The illustration of the wavy microchannels used to isolate EVs. Larger vesicles are directed toward the central output. However, the small EVs (100 nm) are not at the centerline, and they find their way to side outputs. Reproduced with permission from [148]. Copyright 2019 American Chemical Society.

The drawbacks of the conventional strategies include the equipment cost and low efficiency of UC, the risk of contamination and low purity of ultrafiltration and polymer-dependent concentrated methods, and the high labor costs. Additionally, conventional technologies isolate bulk sEVs, while new advances should benefit from the isolation of specific subpopulations of sEVs, such as TDEVs for different downstream applications. All in all, these challenges have resulted in a demand for new strategies. Microfluidics is a great tool to overcome the mentioned challenges because of its ease of processing, simplicity, and high efficiency. Immunoaffinity-based approaches are the most frequent methods used in microfluidic devices [135]. The substrate for isolation can be either magnetic beads or internal surfaces functionalized with antibodies or aptamers targeting EV markers; sEVs commonly express several proteins, including CD9, CD63, and CD81. Devices targeting CD9 [136], EPCAM [136,137,138,139], CD63 [135,138,139,140,141], CD81 [142], phosphatidylserine (PS) [143], and other markers [144] have been developed to isolate EVs. Hisey et al. developed a device using anti-EpCAM and anti-CD9 coated onto herringbone structures to isolate and release EVs from ovarian cancer [136] (Figure 5A). Their results show a nearly five-fold higher yield compared to UC. In a recent work, Kang et al. employed Annexin V (a surface protein) in circular chambers within their ^new^ExoChip device to capture PS positive TDEVs [143] (Figure 5B); PS was shown to be expressed on the outlet leaflet of cancer-associated EVs [143]. The results showed that the isolation efficiency of TDEVs is 90% for annexin V capture compared to 40% for a device conjugated with anti-tetraspanins. A second approach for targeting TDEVs aims to identify an antibody cocktail for EVs from a specific cancer type, i.e., glioblastoma. The ^EV^HB-Chip technology used this method to achieve a low limit of detection of only 100 sEVs/mL while still allowing for sEV release, a critical need in applications such as drug delivery [145]. Although the multiplexed isolation of EVs can be achieved with a combination of antibodies, the process of functionalization has some limitations. Kang et al. addressed this issue by implementing click chemistry in their microchip to isolate sEVs [146]. Briefly, after surface modification of the device with trans-cyclooctene, tetrazine conjugated antibody mixtures were introduced into the microchannels. The isolation of sEVs was facilitated through the inverse electron demand Diels-Alder (IEDDA) interaction between tetrazine and trans-cyclooctene. In addition to specific markers, specially designed microfluidic devices enable the sensitive detection and release of sEVs, which is not possible using conventional immunoaffinity kits. As a workaround to developing release strategies, label-free isolation techniques allow for holistic separation while leaving EVs untouched, making them optimal for functional or drug delivery studies. These technologies rely on size, density, or other physical properties to isolate EVs. For example, Yeh et al. developed a microfluidic chip that consists of multiple zones patterned with carbon nanotubes with different patterning densities to isolate sEVs of different sizes (Figure 5C) [147]. Similarly, inertial microfluidic technologies can isolate particles of differing sizes by using secondary Dean flow within wavy channels to focus larger (300 nm) and smaller (100 nm) vesicles to different channel locations (Figure 5D) [148]. The efficiency and purity of conventional methods are not comparable with the output of this device, which shows an efficiency of 81% and a purity of 92%. Problematically, size-based methods suffer from clogging, require a pure input, and thus, can only be used to separate pure EVs into subpopulations based on size and fail to isolate sEVs from similarly sized particles, such as protein aggregates.

#### 2.2.3. Conventional Methods for sEV Characterization

sEV-related purity is defined by a series of characterizations, including morphology, size, and protein composition [149].The most frequent methods to probe sEV morphology and overall purity are microscopy-based, including atomic force microscopy [150], transmission electron microscopy [151], and field emission scanning electron microscopy [150]. To study the sEV size distribution, optical methods such as dynamic light scattering and nanoparticle tracking analysis are performed [152]. Protein analysis is frequently achieved through antibody-based assays such as ELISA and western blotting [152]. However, many of these methods require sample preparation, prolonged characterization processes, and specialized training. In addition, some lack sensitivity. Outside of determining the presence and purity of sEVs in a sample, common downstream applications of sEV characterization include RNA, DNA, protein, and metabolite profiling. RNA characterization is commonly performed using reverse transcription-qPCR, microarrays, or RNA sequencing. While standard protein profiling techniques are commonly used, increased technological capacities have led to a rise in flow cytometry-based profiling techniques [141]. Other proteomics strategies such as sodium dodecyl sulfate-polyacrylamide (SDS-PAGE) gel electrophoresis followed by liquid chromatography-tandem mass spectrometry (GeLC–MS/MS) [153], and matrix-assisted laser desorption ionization time-off-light (MALDI-TOF) [154,155], have become more prevalent.

#### 2.2.4. New Strategies for sEV Characterization

**Figure 6:**
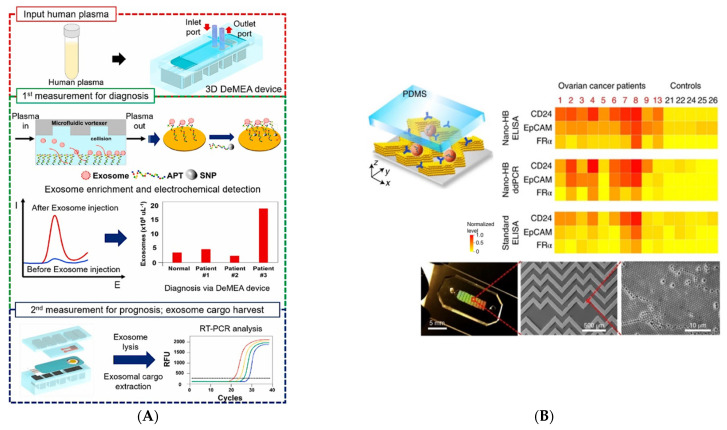

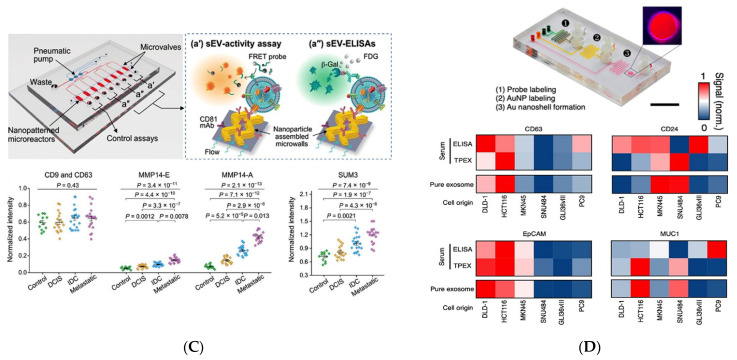
New strategies for EVs’ monitoring and profiling. (**A**) The workflow schematic of the detachable microfluidic device implemented with an electrochemical aptasensor (DeMEA) to quantify EVs. The device in this work uses a herringbone microvortex-generating structure to enhance the isolation efficiency of EVs. Upon the aptamer-mediated capturing of EVs against the EpCAM marker, they can be quantified with the help of a nanocomposite-coated electrode. Reproduced with permission from [162]. Copyright 2020 Elsevier. (**B**) Schematic of the 3D nano-HB device used to detect EVs. With the performance of an on chip ELISA, the heat maps of ovarian cancer EV markers are compared to the conventional ELISA. Reproduced with permission from [142]. Copyright 2019 Springer Nature. (**C**) The diagnostic results of the EV-CLUE chip, which profiles the MMPs expression and activity in EVs from breast cancer clinical samples. From [165]. Reprinted with permission from AAAS. (**D**) The image of the device developed by Wu et al. Results from performed TPEX analysis are compared to conventional ELISA [166]. Reproduced from an open source.

As with ctDNA, there is great interest in the development of microfluidic-based platforms for dual sEV detection, quantification, and characterization. Moving sEV characterization from off-chip to on-chip decreases processing time and sample loss while maintaining high sensitivity. Detection can be accomplished using several methods; recently, approaches based on fluorescent, colorimetric, and electrochemical properties have been reported. For the profiling of EVs, fluorescent labeling is invaluable and has been widely implemented [135,142,156,157]. In addition, SPR has been utilized to quantify captured sEVs [130,158,159]. Notably, a number of microfluidic devices use electrochemical systems for quantification and detection purposes [140,160,161]. For instance, a detachable microfluidic device implemented with an electrochemical aptasensor captures EpCAM^+^ sEVs and generates a signal that translates to sEV quantity, allowing for sEV enumeration on-chip (Figure 6A) [162]. sEV capture on-chip followed by off-chip characterization, including protein quantification, western blot, and miRNA/mRNA analysis, has been common in recent years. Kang et al. developed a single platform for dual isolation of melanoma CTCs and sEVs in conjunction with off-chip RNA profiling. In their device, bean-shaped micro-posts were functionalized with anti-melanoma antibodies. For the first time, this device isolates both melanoma CTCs and EVs from clinical samples simultaneously and allows for multiplexed gene profiling [163]. More recently, Zhang et al. developed nanopatterned 3D herringbone structures (nano-HB) within their microdevice, enabling EV detection with exceptional precision (10 sEVs/μL^−1^) from both ovarian cancer cell lines and clinical samples followed by on-chip ELISA protein characterization to identify sEV phenotypes based on a protein panel (Figure 6B) [142]. This nano-HB structure increases the surface area and alters boundary conditions near the surface, increasing the likelihood of sEV capture. Following isolation, they performed on-chip ELISA for sEV characterization. Early diagnosis of the metastases has a great impact on survivability in patients and an efficient treatment can be selected based on whether it can be detected. Recently, a microfluidic platform consisting of different modules was proposed to profile epithelial–mesenchymal transition (EMT)-related sEVs from breast cancer patients, making the prediction of metastases possible [164]. The sEV profiling from this device evaluates the EMT index, and it results in high sensitivity and specificity in the early detection of cancer metastases. Similarly, the previously developed nano-HB device was also employed to assess the matrix metalloproteinases expression and proteolytic activity within sEVs in clinical breast cancer samples to clarify these EVs’ role in metastasis and monitor cancer progression (Figure 6C) [165]. In a recent work, Wu et al. deployed a novel nanomaterial and microfluidic-based EV characterization technology termed “templated plasmonic for exosomes (TPEX)” (Figure 6D) [166]. In this method, samples are introduced into the device, followed by incubation and dual labeling of samples with fluorescent aptamers and gold nanoparticles. After the development of in situ gold nanoshells, changes in fluorescence intensity and absorbance signal are monitored. For the in situ profiling of exosomal markers, they spiked exosomes derived from six cancer cell lines into human serum and analyzed the expression of CD63, CD24, MUC1, and EpCAM. The output from the TPEX compared to conventional ELISA was shown to be a better match to pure exosomes. Although there are several exciting developments in the detection, quantification, and characterization of EVs, there is still a larger scope to improve the technologies used for the identification of specific TDEVs.

As the scalability and clinical application of these microdevices have been potentiated, including the fabrication procedures of some complex processes such as the nanopatterning of colloidal structures, the addition of micro-level features to them faces some limitations.

**Outlook.** With the advent of a new generation of technologies using micro and nanofluidics and materials, the isolation of sEVs has become notably more efficient [167,168,169,170]. Furthermore, as researchers are exploring the roles of cell type-specific sEVs, the ability to isolate TDEVs is beginning to transform our understanding of their physiological role in a disease setting. Future work is needed to identify cancer-specific markers to isolate tumor cell-specific sEV subpopulations reliably. Following sEV isolation, if robust approaches can be developed to analyze the small amounts of cargos carried by these membrane-bound vesicles, sEVs can be a viable alternative to ctDNA due to their ready availability and stability in circulation. As is the case for ctDNA, continued development of dual on-chip isolation and profiling will pave the way to move sEV analysis to a clinical setting by decreasing processing time and sample loss while increasing reliability.

## 3. Computational Analysis of ctDNA and sEVs

Despite substantial efforts being put into experimental research and clinical studies, there are ambiguities in understanding the fundamental nature of cancer in patients, the interactions in the TME, and the exact relations among cellular species. The emergence of LB technologies and biomarker analysis have tried to shed light on the underlying mechanisms of cancer, and they have improved the diagnosis and prognosis capabilities of the biomarkers. However, there are still some shortcomings when it comes to real-world applications and case-by-case patient assessment. The fast-paced advances in the fields of mathematical modelling and artificial intelligence and their implementation to the oncology field can be powerful tools to overcome the challenges. In the field of mathematical oncology, the complexities in signaling pathways and the number of cells and cytokines in the TME can be scrutinized to fully understand them; especially when studying the effects of various treatments, the accurate correlations between TME’s compartments can be comprehended and a delicate balance of treatments can be studied.

### 3.1. Computational Models for Studying ctDNA

Computational methods are utilized to improve the sensitivity of the digital PCR for LB using the elimination of background artifacts that can interfere with the detection of rare ctDNA [171]. They are also vastly used for sequencing [172] but these techniques are not specific to plasma DNA and are out of the scope of this review. Here, we focus on mathematical models that are developed to understand the relationship between tumors and ctDNA shedding. For instance, stochastic differential equations are utilized to investigate the correlation between the tumor size and ctDNA levels in the bloodstream. The results suggest that it takes several years for a tumor seeded from a single cell to become as large as an olive and detectable by the conventional imaging techniques, while ctDNA could be detected up to 160 days earlier [173,174]. Kinetics modeling also suggests that ctDNA levels could distinguish between aggressive and non-aggressive tumors much earlier than regular imaging methods [175].

In a recent study, the minimum size of a tumor for both early and relapse detection is determined by modeling the shedding probability of ctDNA into the bloodstream [173]. In this study, available data of lung cancer patients are used to estimate parameters related to the cell lifecycle. It was first assumed that cells release their nucleic acid content to the bloodstream only during apoptosis. In turn, a close simple equation was derived that could predict the concentration of ctDNA in blood:

$$C=M.d.q_{d}/\left(\varepsilon +r\right)$$

In this equation, *d*, *r*, *q_d_*, *M*, and *ε*, respectively, denote the cell death rate, the tumor net growth rate (birth rate minus death rate of cells), the shedding probability during cells’ apoptosis, the tumor size, and the ctDNA elimination rate. Particularly, *q_d_* is a critical parameter and was calculated to be 1.4 × 10^−4^ hGE (haploid genome equivalents) per cell death for cancerous cells. The shedding rate ($d.q_{d}$) for lung benign cells was also calculated to be 9.8 × 10^−6^ hGE per cell per day. According to this research, the shedding rate is directly proportional to the size of the tumor. The model eventually predicts that the size limit for the detection of a tumor using ctDNA assays is 2.1 cm, which shows 40% improvement in comparison with the current state-of-the-art technologies that have a detection size limit of 3.5 cm, provided that the test is carried out on a yearly basis.

There is also evidence that tumors inside a body shed DNA in different rates while the mechanisms are not yet fully understood. In addition to the tumor size, the molecular profile [176], degree of resistance against medications [177], and type of the cancer [178] are reported as the other possible underlying reasons. One study attempted to derive a model to describe relationship between influential variables. More than 6000 cases were studied and it was substantiated that the more mortal a cancer, the more likely it is to shed DNA molecules [178]. However, in this study, multiple tumors (primary and metastatic tumors) were not compared, and the results were from different individuals. In case of a metastatic cancer, multiple tumors could shed into the blood simultaneously, so tumors may not contribute equally to shed cancerous DNA content in the blood. A mathematical model was recently introduced to determine the relative contribution of any tissue in the collected cfDNA sample and was applied to study gastrointestinal cancer patients who had multiple clusters of tumors spread in their body [179]. The modeling outcomes were verified with the autopsy results. The results indicated that the progressing tumors were likely shedding the majority of blood circulating DNA. This claim suggests that mutations with higher frequency could be a possible novel mechanism of drug resistance because these tumors have resisted the applied treatment. However, the data relevant to this model are limited and larger numbers of experiments are yet required.

### 3.2. Computational Models for Studying sEVs

**Figure 7:**
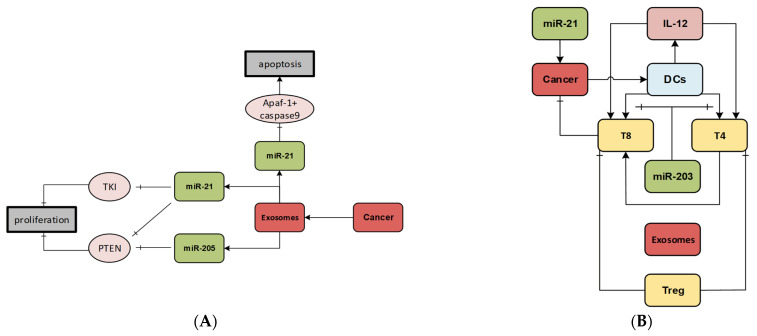

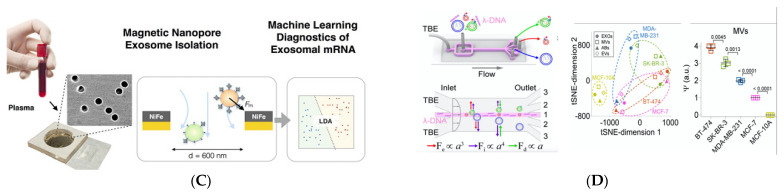
ML applications and mathematical modeling of ctDNA and EVs in cancer. (**A**) The pathway modeled by Lai et al. In this model, EV miRNAs can act as effectors on different pathways, altering apoptosis and proliferation of cancer cells [183]. Adapted from an open source. (**B**) The cellular network model tries to explore the effects of EV miRNAs 21 and 203 in pancreatic cancer. This model embodies more subtypes of immune cells, such as regulatory T cells, T helper type 1 cells, and cytotoxic CD8+ cells. Reproduced with permission from [184]. Copyright 2017 Springer Nature. (**C**) The ExoTENPO device developed by Ko et al. to isolate EVs uses an ML classifier to distinguish different cancer biomarkers in various stages. Reproduced with permission from [196]. Copyright 2017 American Chemical Society. (**D**) The schematic of the microfluidic device integrated with an LDA classifier to discover an LB biomarker for breast cancer. Reproduced with permission from [198]. Copyright 2019 American Chemical Society.

Several studies performed in silico analysis of sEVs’ role in the TME to understand cellular communications through different sEV contents, monitor their contribution in cancer evolution, and finally evaluate cancer treatment methods. A model that consists of cancer, dendritic, and other killer cells investigates the interplay among cancer and immune cells [180]. In this framework, simplified non-sEV- and sEV-based interactions among cells were modeled using nonlinear ordinary differential equations. With comparison of these two models, the appearance and dynamics of different cancer states were studied. The inclusion of sEV-based interactions between dendritic cells and cancer cells in the model results in an intermediate level of cancer stage (a moderate cancer load and a high-level immune response). This stage plays a critical role in assessing the effect of different cancer therapy regimes. The developed model reveals that radiation therapy, by itself, cannot lead to a low or intermediate-level cancer state, although it decreases the tumor load. This model has been adopted to simulate a combined radiation/DC-immunotherapy treatment. With three sets of consecutive alternating treatments, radiation therapy followed by immunotherapy, a high-level cancer can be transitioned into an intermediate-level cancer without excessive radiation or immune-boosting doses. Ultimately, different simulation shows that standalone DC-immunotherapy can be highly effective in inducing an intermediate- to low-level transition. sEV miRNAs have shown multiple effects in the TME and have diagnostic value (reviewed thoroughly in [181,182]), and different mathematical models have been produced in attempts to focus on their effects. One model elucidates the role of sEV miRNAs 21, 205, and 155 on tumor growth via the Ras-Raf-MEK-ERK and the PI3K-AKT signaling pathways (Figure 7A) [183]. The obtained results suggest that sEV miRNAs have a significant role in the expansion of NSCLC cells, and furthermore, the administration of anti-miRNA 21, 205, and 155 drugs can inhibit tumor growth. A more physiologically relevant model was then developed by including additional cell types, such as CD4+ T helper type 1 (Th1) cells, regulatory T cells, cytotoxic CD8+ cells, and DCs, to assess the role of EV miRNAs in pancreatic cancer (Figure 7B) [184]. They employed partial differential equations to quantify the relationship between cancer-released sEV miRNAs 21 and 203 and tumor growth. In addition, a model was developed that accurately correlates an increase in the bulk number of sEVs to tumor volumes in human cancer cases [185]. In this study, after the embedding of data from preclinical mouse cancer models into the mathematical models, and the optimization of these data, they successfully predicted the relation between tumor size and numbers of circulating sEVs and the fold change increase in the number of bulk sEVs as a function of tumor volume in human cancer cases. Treatment of cancer-stricken patients with chemotherapeutic drugs may cause resistance to the administered drugs. The mechanism by which this might occur is of a great interest. The role of sEVs as a chemoresistance-inducing factor was also studied and [186] hypothesized that one of the contributing factors to drug resistance is the oncogenic sEVs in the TME, which can act as mediators of turning sensitive cancer cells into resistant ones. Their results suggest that presence of sEVs can increase the population of resistant cancer cells when a low dosage of drug is applied. However, in case of high dosages, the opposite results were observed since the number of cancer cell-producing sEVs is reduced; although this is a desired result, high dosages can have other side effects.

### 3.3. Machine Learning Approaches for Cancer Screening

With the improvement in oncology-related diagnosis, imaging, and drug discovery technologies and platforms, large data sets are being generated. These data can be categorized into different genomic, proteomic, and radiomic subcategories. Artificial intelligence, including machine learning (ML) and deep learning (DL) methods, have been implemented for various applications including biomarker discovery, and digital pathology (review [187,188,189,190,191]). In the case of cancer biomarkers, DL approaches have been utilized to detect ctDNA markers in cancer cases [192,193]. Additionally, ML algorithms have the potential of validating EV markers for cancer diagnosis considering various EV-contents such as long RNAs in pancreatic ductal adenocarcinoma [194], or wide-ranging arrays of novel proteins other than pan-EV proteins in multiple cancers [195]. The integration of ML/DL algorithms with microfluidic devices can be leveraged to develop platforms with higher diagnosis value. The exosome track-etched magnetic nanopore (ExoTENPO) chip is formed of membranes coated with a magnetic layer, and with nanopores with a size of 600 nm (Figure 7C) [196]. Following the immunomagnetic isolation of pancreatic cancer sEVs (from KPCY mice) with the ExoTENPO chip, they were lysed on-chip, and qPCR data of EVs’ mRNA contents were analyzed for an array of eight genes including *ARG1*, *CD63*, *CK18*, *Erbb3*, *GAPDH*, *KRAS*, and *ODC1*. Based on an ML algorithm, linear discriminant analysis (LDA), the data can be used to subcategorize samples into healthy, pancreatic intra-epithelial neoplasia lesion, and cancer cases. This classification was also verified for clinical sample. To demonstrate the ability of this device and the classification of their ML algorithm, the same research group analyzed EV-miRNA signatures to diagnose pancreatic cancer [197]. In another study, a microdevice was designed to isolate sEVs with different sizes, namely, exosomes, microvesicles, and apoptotic bodies [198]. The isolation could occur with the help of a viscoelastic fluid and Newtonian sheath flow within the device (Figure 7D). With a different approach from previous studies, the authors studied two markers, HER2 and EpCAM, in sEVs with different sizes to check if different subtypes of sEVs could have a different diagnostic ability. A similar ML approach was developed to classify the data from cell lines as well as clinical ones from cohort of stage II breast cancer patients. The results suggest microvesicles can be a better LB biomarker since they tend to show better scores for LDA and t-distributed stochastic neighbor embedding analysis.

**Outlook**. All in all, these mathematical models can be used with patients’ clinical data to help oncologists narrow their focus and adopt a thoughtful and personalized set of therapies for their patients. The model developed by Lu et al. [180] has the potential to personalize cancer therapy; however, the downside of this work is that the interaction among immune cells and cancer cells has not been fully included, and it can be improved by implementing more specific intercellular communications. Models containing sEV-miRNAs provide us with useful clues about the TME; however, there is a need to investigate more sEV contents, such as long non-coding RNAs and heat shock proteins, to come up with new therapies. Artificial intelligence’s integration with oncology-related subfields offers insightful information about patients and the TME, and makes it easier for scientists and physicians to decide about a therapy selection, drug, and biomarker discovery method in a more unbiassed manner.

## 4. Conclusions

LB biomarkers represent a means to profile tumors non-invasively and may aid in early diagnosis and prognosis, as well as the molecular characterization of disease and the monitoring of treatment efficacy. Compared to the traditional tissue biopsy techniques, LB testing is rapid and more useful when a tumor’s location makes tissue biopsy unfeasible. With the help of new advances, the isolation and characterization of LB biomarkers can be more cost-effective compared to tissue biopsy CTCs, and ctDNA and EVs play complementary roles in cancer progression; thus, a comprehensive understanding of the disease complexity requires a multiplexed analysis of these circulating biomarkers. Although many new technologies and computational models have been developed for the detection and characterization of ctDNA and EVs, the majority have yet to demonstrate clinical utility. We envision that rigorous validation and large cohort clinical trials will allow these biomarkers to transform cancer care.
